# "The Hospital" Nursing Mirror

**Published:** 1901-05-25

**Authors:** 


"The Hospital" Nursing Mirror.

Being the Nursing Section of "The Hospital."

[Contributions for this Section of "The Hospital" should be addressed to the Editor, "The Hospital" Nursing Mirror, 28 & 29 Southampton Street Strand, London, W.C.]

### Notes on News from the Nursing World.

### THE COLONIAL NURSING ASSOCIATION.

The fifth annual report of the Colonial Nursing Association, which was adopted at the annual meeting on Wednesday, shows that the number of nurses sent out in the twelve months ending April 30th, 1901, was 29 as against 26 in the year ending April 30th, 1900. The total number of nurses now at work is 67, of whom 46 are employed in Government hospitals and 21 as private nurses. The only new branch started was in Costa Rica; the nurse was sent out in August and has given great satisfaction. The chief feature of the year has been the great increase in the staff of nurses employed by the Government on the West Coast of Africa. Eight fresh nurses were supplied to Nigeria, and Old Calabar applied for a second nurse to supplement the one sent out last year. Two extra nurses were also selected for the hospital at Singapore, bringing the staff there up to eight. "Hitherto," says the report, "all Government appointments have been made at the request of the Colonial Office; this year the Foreign Office has also made use of the organisation of the Association to supply two nurses for work in Zomba, British Central Africa." This is a compliment to the Executive Committee of the Association, and we see no reason why the Foreign Office should not make use of it whenever it is in want of nurses. Mention has already been made of the fact that Matron Ward and Sister K. Nevill, who were sent out by the Association, received the Royal Red Cross in recognition of their services at the base hospital during the late Ashanti campaign. The foundation of the sick pay fund, to provide temporary financial assistance to any nurse who may become incapacitated during her connection with the Association, and the public start of the Scottish branch, have been postponed to a more opportune time, "when greater monetary support might be looked for." Earl Grey, who took the chair at the meeting, has succeeded the late Lord Loch as president, and Mrs. Francis Piggott has been succeeded as honorary secretary by Mrs. Ernest Debenham. Happily Mrs. Piggott, to whose energy and initiative (as the report observes) the organisation owes its establishment, is able to continue her work on the committee, and to direct, as chairman of the Nursing Committee, the important work of the election of nurses. She has also been elected honorary vice-president as a recognition of the place in its counsels to which not only all members, but all who are in the least acquainted with the history of the Colonial Nursing Association, must feel that she is entitled.

### THE CONDEMNATION OF THE IRISH BOARD OF GUARDIANS.

In another page will be found the substance of the evidence given before the Judicial Committee of the Irish Privy Council in support of the Nursing Order of February 4th, 1901, which has been appealed against by 159 unions in the sister island. No one who reads the testimony of the inspectors, ex-inspectors, and eminent medical men who were called on the occasion will be in the slightest doubt as to the deplorable condition of things in the workhouse infirmaries. While the Judicial Committee came to the conclusion that the order must be disallowed on the ground that there was no evidence to justify the general revocation of Articles 24 and 39 of the order of 1882, they recognise the necessity "for some rule or order which will make better provision for efficient nursing." There should be no delay in framing such an order. The inability, or the indisposition, of the Irish Boards of Guardians to deal with the question has been proved up to the hilt.

### NURSING ASSOCIATIONS AND THE EDUCATION BILL.

At the annual meeting of the Rural and Urban Trained Nurses' Society for Derbyshire, Lady Laura Ridding, who presided, referred to the question of affiliation with Queen Victoria's Jubilee Institute. On a former occasion an application of the Society to the Institute for affiliation was refused, but the Council having now proposed to reconsider their decision and to admit county and rural associations under certain conditions, Lady Laura suggested that the proposal should be made again. Eventually the matter was referred to the executive committee to report upon at a future meeting. The president, in this connection, touched upon a point of general interest. She observed that in Derbyshire and Nottinghamshire, as well as in other parts of the country, nursing lectures and grants came through the medium of the County Council. "It was, however, just possible that greater educational powers would be given to the County Councils, in which case the nursing associations would lose their grants. It would then become a question of where should they get their grants from? and that was why she urged their affiliation with the Q.V.J.I." Lady Laura, of course, referred to what might happen in the event of the new Education Bill becoming law. If, in consequence, nursing associations are to lose their grants, they will not be anxious that it should pass.

### "BART'S" EXTENSION.

After all, St. Bartholomew's will probably succeed in obtaining part of the site of Christ's Hospital, which adjoins it, when the Bluecoat School removes to its new quarters at Horsham. It is proposed to use some of the annexes for new quarters for the nurses, who are at present scattered over different parts of the hospital buildings, some of which are in a rather dilapidated condition and unsuitable for their purpose, having formerly been old houses (situated in Little Britain), which have been knocked together in their present form. No doubt the new nurses' sitting-room, though it could scarcely be prettier, will be arranged with a view to giving more room for the game of "Ping-Pong," which the matron has recently presented to the nurses. The nurses' library also has grown so much of late years that it will need further accommodation for its bookshelves. Here all grades of the nursing staff, from the assistant-matron down to the newest probationer, may meet on equal ground to read, write, study or rest, for which ample opportunity is given in the Rule of Silence, and the deep armchairs.

### THE NURSING QUESTION AT BRISTOL WORKHOUSE INFIRMARY.

At the last meeting of the Bristol Board of Guardians, the hospital committee presented their report in reference to the application of the medical officer at Eastville Workhouse for two more probationer nurses. It appears that the complete staff consists of 22 nurses, including the superintendent nurse and the night superintendent, who take no actual share of the work excepting supervision, leaving 20 nurses engaged as follows:—Four male wards, about 100 inmates, six nurses engaged by day and three by night; three female wards, about 80 inmates, five nurses by day and two by night; in the children's ward, about 29 inmates, three nurses by day and one by night. Owing to two of the wards, in the body of the workhouse, now being used for convalescent cases from the male hospital, the removal of these patients having been arranged by the board to meet the pressure for acute cases, the hospital wards for men are filled with patients requiring more skilled and individual attention. The female wards remain more or less full throughout the year, and require, the committee affirm, even more attention than the males. There are about 80 occupants, and of these 17 are completely paralysed and incontinent, and consequently need attention every two hours. The remainder are nearly all bedridden. The night nurses of late have not been able to finish their own work until about 9 A.M., being an hour later than the proper time; and the day nurses, especially in the female wards, have frequently been unable to take any time off duty. In these circumstances, the committee approve the proposal to engage one more probationer for day duty, and one for night duty. Nor was an attempt to postpone the consideration of the matter on the ground that "some members desired that the nurses should have longer holidays" successful. We hope that the appointment of the additional probationers will enable the committee to extend the summer holidays of the nurses; for it is very clear that they are needed.

### A NEW DEPARTURE AT CORK INFIRMARY.

A special meeting of the joint committee of manage- of the Cork South Charitable Infirmary and County Hospital was held last Thursday to consider a notice of motion by Mr. Howard to the effect that the vacancy for a matron be filled by a member of a religious order. The Bishop of Cloyne presided, and there was a very large attendance. Mr. Howard proposed his motion, which was seconded by Mr. A. Roche, and supported by several Roman Catholic members. An amendment was moved by the Right Rev. Dr. Meade, the Protestant Bishop of Cork, Cloyne, and Ross, "that the usual advertisement inviting candidates for the vacancy should be issued." On a division, Mr. Howard's motion was carried by 15 to 7. This is a new departure on the part of the committee. The late matron, Miss L. M. Moss, was trained at Crumpsall Infirmary, and she had the assistance of a staff superintendent nurse who was also trained at Crumpsall. The appointment of a member of a religious order unless she has undergone a full training is a retrogressive step, and we are not surprised to learn that it has excited a great deal of comment among trained nurses in Cork.

### HEROISM OF A NURSE AT STAFFORD.

There is no doubt that the serious fire at the old infirmary of Stafford Workhouse last week, which resulted in the loss of seven lives, and much damage to property, would have been still more disastrous in its consequences if it had not been for the bravery of Miss Langatreen, the nurse in charge of the imbecile wards. Between one and two in the morning one of the inmates came to the nurse's room, and gave the alarm. The nurse immediately went down the passage and heard the cook calling "fire." On opening the door of the corridor leading to the cook's room she was forced back by the smoke and heat. Finding she could not proceed further she told the occupants of the wards to get up and run to the new wards in the main block. She also fetched three women and their babies out of another ward. Then she got the fire-hose to work, and began to play it on the burning part of the building. She continued to work the hose upstairs and down till she was relieved by the matron and laundress, by which time she was naturally quite exhausted, the force of the water taxing all her strength to hold the hose. But thanks to her gallant efforts, the flames were prevented from spreading to the main block containing 260 persons. It is the universal opinion that a great calamity was thus averted, and Miss Langatreen has been warmly and deservedly congratulated upon the courage she displayed.

### THE MATRONSHIP OF MACCLESFIELD INFIRMARY.

Several correspondents have written us, though not for publication, that our remarks last week as to the qualities desirable in the new matron of Macclesfield Infirmary suggested that the retiring matron is not a lady of ripe experience, nor devoted to her work. We cannot understand how this interpretation could have occurred to any one who is familiar with the nursing career of Miss Noble. Trained at the Children's Hospital, Nottingham, and the Royal Infirmary, Bristol, she was appointed sister at the Children's Hospital, Bradford, in 1889, becoming in 1892 head nurse at the General Infirmary, Northampton. In 1893 she was invited, as sister in charge, to inaugurate the Chorley Cottage Hospital, where she remained until 1896. Having placed the Chorley Hospital on a thoroughly good footing, she accepted in that year the post of matron of Macclesfield Infirmary, where she has been very popular among the nurses, servants, and patients. For the benefit of the nurses she started a weekly social evening in her own sitting-room for such as could attend, and on her return from her holiday last autumn the servants made a presentation to her. From all we can gather, the House Committee will be fortunate if Miss Noble's successor is equally well qualified and capable.

### MISS BAXTER'S VALEDICTION.

A memorial, signed by over 400 persons, was presented to Miss Baxter, formerly matron of the Women and Children's Hospital, Cork, wishing her to establish a nursing institution either in Cork or the neighbourhood, but while thanking the memorialists for their flattering invitation, she has felt herself compelled to decline it. In the course of her reply Miss Baxter enters into details respecting her work in Cork, and the circumstances which led to her resignation. As to the former, the value of her services, extending over fourteen years, has been widely acknowledged; and as to the latter, we do not think that any useful purpose can be answered by referring to them further. We prefer to rejoice at the admirable tone observed by Miss Baxter in her closing remarks, which explain her refusal to open a nursing institution in Cork. "I have given," she says, "the Cork Women and

Children's Hospital too much of my work to even seem to enter into unfriendly rivalry with it. A hospital exists not for its committee or its lady patronesses, but for the sick and suffering who find relief within its walls. With them I can have no quarrel, and any passing sense of grievance is a small matter indeed compared with the satisfaction of which no one can deprive me of knowing, namely, that for long after my poor name is forgotten, suffering women and helpless children will, by God's mercy, be somewhat the better of my connection with the Cork Women and Children's Hospital."

### MIDWIVES AND DEATH-CERTIFICATES.

Why will midwives give certificates of death when they know they have no legal right to give a certificate of anything but a still-birth? Of course, the present arrangements are unsatisfactory, and the line is drawn thick or fine according to individual conscience between still-birth and death soon after delivery; but in the case which came before a coroner's jury the other day, the child was five days old, and the midwife, who is attached to a hospital lying-in charity, could have no possible reason for giving herself the trouble and risk of granting a certificate, albeit she was authorised to do so by the resident obstetric physician at the hospital.

### METROPOLITAN ASYLUMS BOARD.

At the first meeting of the newly-elected Metropolitan Asylums Board on Saturday, the children's committee submitted a revised wages scale for the staff for the Bridge School, Witham, the number of children being 160. The chief difference is the increase of the nursing staff. It was not known at first how much treatment the heads of the children would want, but as the dermatologist requires each child's head to be treated twice a day, the committee recommended that the number of charge nurses, assistant nurses and keepers be increased in each case by two, and that the revised scale of salaries be as follows:—Charge nurses, £30, rising £2 per annum to £36; assistant nurse, £20, rising £1 per annum to £24, with board, lodging, washing, and uniforms. The recommendations were adopted.

### A PATTERN OF PROGRESS.

At the annual meeting of the Harrogate District Nursing Society Viscount Mountgarret, who was among the speakers, said that in his opinion "it required but a little effort to make the society a pattern of progress to the whole of England." In other words, the only need of the organisation is more money to enable the committee to pay the salary of the third nurse whom they have had to engage in order to cope with the increasing work. More money is, we fear, a general want, and it is satisfactory to find that the nursing movement is popular in Harrogate, and that the relations between the district nurses and the members of the medical profession are cordial. We fancy, however, that there are a number of nursing associations which might already claim to be patterns of progress.

### THE DISTRICT COUNCIL AND THE NURSING CUPBOARD.

The courteous manner in which the Redruth District Council acceded to a request of the hon. secretary of the Redruth Nursing Association for permission for space for the cupboard containing nursing appliances and requisites, in the hall of the District Council rooms, deserves to be recorded. The cupboard, which was procured for a Cornish nurse having at the time a house of her own with plenty of spare room, proved to be rather a white elephant to the present district nurse in lodgings, hence the application to the District Council, whose chambers in a central position are commodious and more than sufficiently ample for official purposes. In bringing forward the application the District Council chairman spoke of the valuable services rendered by the district nurse to residents of all classes in the town and neighbourhood, his statements being concurred in and confirmed by other members of the Council, one of whom said that if the Council refused permission he should personally provide a place for the cupboard. On the other hand, a member urged that there was a possibility of infection being spread by articles lent to persons suffering from infectious diseases being stored there. He was promptly squashed by the medical officer, who showed that a rule of the District Nursing Association prohibited the nurse from attending infectious cases. Finally, permission was given, there being only a single dissentient.

### THE CANCER HOSPITAL, BROMPTON.

The outbreak of fire which occurred at the Cancer Hospital, Brompton, early last Friday, fortunately for all concerned, was promptly put out by the porter assisted by the lady night superintendent with the help of three brave soldiers and a friend, before the arrival of the fire brigade. The nurses were the only persons disturbed, most of the patients being quite ignorant of any danger.

### AMUSING EXPERIENCE OF A BOARD SCHOOL NURSE.

At the annual meeting of the South London District Nursing Association on Saturday, Lord Stamford, Dr. Hayward, and Miss Alice Hughes, dwelt on the importance of the duties of a skilled nurse who is entrusted with the task of tending the Board School children. Canon Erskine Clarke, who also said he was satisfied from personal observation as to the usefulness of the work, told a delightful story, to illustrate his observation that one of the joys of childhood is to like many things that grown-up people dislike, and that the school children who are seen by the nurse very much enjoy being treated for small ailments. "He knew of a small boy who was found by the nurse sitting in the midst of a crowded street in Battersea, and who said, in answer to her inquiries, 'Please I'm waiting to be run over.' That boy had heard of the delights of being in hospital."

### SHORT ITEMS.

At a meeting held in the Council Chamber, Dublin Castle, on Monday afternoon, under the presidency of Countess Cadogan, it was resolved to raise an endowment fund to the memory of Queen Victoria on behalf of the Queen Victoria Institute for Nurses. Catharine Duchess of Westminster, Lady Mary Howard, Lady Elizabeth Biddulph, and Viscountess Baring have joined the Memorial Committee.—According to the latest progress reports from South Africa, Nursing Sisters Edwards and Richardson are still dangerously ill, but Sister Higgs is much better.—Nursing Sister C. Harvest has been invalided home and is returning in the s.s. *Bavarian.*—The summer sale of the Working Ladies' Guild will be held at 2 Carlton House Terrace, by permission of Lord and Lady Hawkesbury, on June 5th and 6th.—The report of the After-Care Association for Poor Persons Discharged Recovered from Asylums for the Insane shows that during the past twelve months the council had before them the cases of 132 women and 63 men. Many were assisted by being boarded out in the cottages in the country, by being given grants of money or clothing, and by finding occupation for them; but others it was found impossible to help.

### Lectures to Nurses on Medicine and Medical Nursing.

By NORMAN DALTON, M.D. London, F.R.C.P., Physician to King's College Hospital.

### LECTURE XI.—THE SYMPTOMS OF DISEASE OF THE MITRAL VALVE. EMBOLISM.

By listening to the heart's sounds physicians can usually discover which valve of the heart is diseased; but, in addition, the symptoms presented by disease of the mitral are different from those presented by disease of the aortic valve.

*Symptoms of Mitral Disease.*—If the defect in the valve be "compensated" (see last lecture) no symptoms occur, although the physician can detect the condition by listening to the heart. In such cases he merely tries to maintain compensation by ordering nourishing food and strengthening medicines, such as iron, arsenic, &c. Moderate exercise is good, but all violent forms of exertion must be given up. Mental worry is injurious to all affections of the heart. Nurses are seldom called in for "compensated" cases; but if they should be they can only take the usual precautions against exposure to cold, and watch for any symptoms such as shortness of breath, cough, &c., which may indicate that compensation is failing. In particular they must look out for the occurrence of dropsy in the feet.

When compensation fails, the symptoms of "back pressure" set in; that is those symptoms which are produced by habitual venous congestion all over the body. The earliest of these is shortness of breath on exertion. The meaning of this symptom is that there is still enough compensation present while the heart is doing its ordinary work; but that, as soon as an effort is made and the heart has to work harder, it is unequal to the extra strain; and consequently the blood becomes dammed up in the lungs, the body as a whole does not get enough oxygen, and a sense of shortness of breath is experienced. When compensation entirely fails and the heart becomes dilated, the back pressure symptoms become constant. Œdema sets in in the feet, rises to the legs and thighs and finally to the peritoneal cavity. The congestion of the lungs is now permanent, so that shortness of breath is present even when the patient is at rest; and finally it becomes so bad that the patient cannot breathe unless he sits up, a condition which is called "orthopnœa." There are two reasons why it is easier to breathe when sitting up than when lying down. Firstly, when lying down, some part of the chest wall must be pressed against the bed and consequently the whole chest cannot expand as fully as it is capable of doing; and secondly, when sitting up the extraordinary muscles of inspiration can be used with greater effect, particularly those muscles like the pectorales and the serratus magnus which are attached to the chest at the one end and to the arm and scapula at the other. Under ordinary circumstances these muscles are used to move the upper limb and the shoulder, in which case the chest is the fixed point from which they act. But when we wish to use them for the purpose of breathing we fix the arm and shoulder by holding on to some firm support, and so make them the fixed points, so that the result of the contraction of the muscles is to raise the ribs and help the inspiratory movements. It is obvious that the fixation of the arm and shoulder is much easier in the sitting position, and many persons when suffering from dyspnœa not only sit up but lean forward and press their elbows on the arms of a chair or on a table in front of them so as to fix the shoulders. If the congestion of the lungs lasts any length of time, the lung becomes œdematous, that is, the serum gets out of the capillaries and enters the air vesicles. The patient then begins to cough in order to bring up this serum. But in many cases bronchitis sets in and the bronchial tubes become filled with mucus, the sputum becomes thick and tenacious, and the cough and shortness of breath become worse. Sometimes even the red blood corpuscles escape from the capillaries into the air vesicles, and then the sputum contains blood. It must be remembered, however, that the hæmoptysis in cases of mitral disease may be due to embolism in the lungs. In some cases pneumonia supervenes in the congested lungs, and then the temperature becomes high. In an uncomplicated case the temperature is usually subnormal, so that the presence of fever means either that an inflammatory complication such as pneumonia or pleurisy has occurred, or that embolism has taken place, or that the affection of the heart has become septic.

As the term embolism has been mentioned twice, it is as well to explain its meaning now. When the endocardium becomes inflamed, the blood, as it passes over the inflamed spots, deposits small pieces of blood clot on them. These pieces of clot are much more commonly found on the valves than on any other part of the endocardium, except in cases of septic endocarditis. On the valves the clot forms little projections which are called vegetations, and sometimes the vegetations break off and are carried by the blood stream into the aorta and thence into one or other of the arteries. The vegetation which is thus carried along in the blood is called an embolus. It is obvious that the embolus will be carried along some artery until it reaches an arteriole which is too small to allow it to pass. Then it completely blocks that arteriole and the piece of tissue (which may be situated in the lungs, spleen, brain, &c.) which is supplied by that arteriole can no longer get any arterial blood. Consequently the function of that piece of tissue is immediately stopped and it usually soon degenerates. If the embolus contain septic germs, as is the case in septic endocarditis, an abscess forms in the organ, at the spot where the embolus became impacted. It must be understood that there are other kinds of emboli besides the pieces of clot which come from the heart.

To return to mitral disease: in all bad cases the lips and fingers are blue, and the pulse is rapid, weak, and small. Sometimes the heart can be felt to be beating strongly while the pulse at the wrist can scarcely be felt. The liver becomes large and slight jaundice may be present, while the urine is scanty and dark coloured. In some cases the congestion of the stomach leads to nausea and vomiting. As the case becomes worse the patient is unable to sleep. The reason of this is that the extraordinary muscles of respiration are voluntary muscles; that is they only act when the patient makes them do so by the exercise of his will, and they cease to act during sleep. Consequently, as soon as the patient doses the involuntary muscles of respiration (the diaphragm, &c.) are left to carry on the breathing alone, which they cannot do, so that the patient, in his sleep, feels a horrible sense of suffocation which wakes him at once and impels him to start working the extraordinary muscles again.

### To Nurses.

**WE invite contributions from any of our readers, and shall be glad to pay for "Notes on News from the Nursing World," or for articles describing nursing experiences, or dealing with any nursing question from an original point of view. The minimum payment for contributions is 5s., but we welcome interesting contributions of a column, or a page, in length. It may be added that notices of entertainments, presentations, and deaths are not paid for, but, of course, we are always glad to receive them. All rejected manuscripts are returned in due course, and all payments for manuscripts used are made as early as possible after the beginning of each quarter.**

### The Colonial Nursing Association.

ANNUAL MEETING.

The ball-room at Londonderry House was well filled on Wednesday afternoon last, when the annual meeting of the Colonial Nursing Association took place.

Earl Grey was in the chair, and before asking Lord Onslow to move the adoption of the annual report he reminded the meeting of the beginnings of the association. It owed its origin to the painful impression left on the mind of Mrs. Piggott some few years ago in Mauritius, when three young Englishmen died, one after the other, in the prime of life, mainly for want of nursing. Mrs. Piggott appealed to Mr. Chamberlain at the Colonial Office, and the result was the formation of the Colonial Nursing Association. The Association had a strong supporter in the president, Lord Loch. No one realised more fully than he did how useful the work might be. His death was a very great loss. Speaking personally, Lord Grey said he never felt prouder of being an Englishman than when he thought of the numbers of young Englishmen who were ready to go anywhere and to run any risk if they thought they could promote the well-being of the Empire. The business of the Association was to prevent, as far as possible, the waste of material in the unhealthy climate to which it was often necessary to send them. It aimed at paying the nurses a minimum salary of £60 apiece, paying their passage, and making temporary advances when necessary. A short time ago Mr. Chamberlain, to whom the Association owed a debt of gratitude, issued an appeal for £5,000. He was glad to say that £3,000 had already been contributed, and he hoped that the result of the meeting would be to provide the balance. Since coming into the room he had received a cheque for £21 from Sir John Holder, who was unable to be present, and Lord Crawshaw had contributed £5. He hoped that example would be followed.

Lord Onslow then moved the adoption of the fifth annual report. He congratulated the Association on being fortunate enough to keep Mrs. Piggott as honorary vice-president and as chairman of the Nursing Committee, in which capacity she carried out the difficult task of selecting the nurses for sending to the Colonies. It was often said that the two kindred sciences of medicine and surgery had not advanced with equal strides, but everyone would agree that the nursing of the sick had made a gigantic advance. It was largely owing to this that the existence of the Colonial Nursing Association was due. The status of the nursing profession had greatly changed during the last fifty years, and a large number of educated and refined women were now eager to devote their lives to this work. There was no lack of capable and willing ladies, but there was a lack of funds for sending them out. Wherever the English tongue was spoken, it ought to be possible to send a trained nurse. From his experience in the Colonial Office he could say that, although there was only a limited number of posts at their disposal, and many of these in places where the climate was deadly, there was never any lack of applicants to fill them. He believed there would soon be a large emigration of Europeans to the West Coast of Africa, and it was absolutely necessary that proper medical and surgical facilities, and, above all, proper nursing, should be made possible there. If England was to take up the white man's burden, it must see that the ill effects of climate were properly guarded against.

Sir Cuthbert Quilter seconded the adoption of the report. He spoke on the financial aspect of the Association, and drew attention to the recent action of some of the steamship companies in withdrawing their concessions as to the passages of nurses; the P. & O. had been the first to do so, and their example had been followed by the North German Lloyd Line. He hoped his words would reach the steamship companies; no doubt the burden of the coal-tax was heavy, but it seemed hard that it should be visited in this way on the nurses, whose work everyone acknowledged to be so useful to the Empire.

Colonel Sir James Willcocks supported the motion. He had had personal experience of the work of the nursing sisters on the West Coast of Africa; the climate was so unhealthy that a patient was frequently obliged to return to hospital soon after being discharged. A non-commissioned officer under his command had once applied to be sent down to the base hospital, thinking he would certainly recover under the care of the nursing sisters. The journey was a long and difficult one, but the man was very anxious to go, and leave was granted. Two months later, he himself saw the officer; he had quite recovered. His remark was: "Sir, I told you so." There were seven nursing sister in Ashanti, and anyone who knew the conditions of living on the West Coast would realise the value of their work. He was very pleased to say that the devotion of the matron, Miss Ward, and Nursing Sister Nevill, in volunteering to stay another year when their time had expired, had been rewarded, by his Majesty the King, on Mr. Chamberlain's recommendation, with the Royal Red Cross.

Lady Balfour of Burleigh supported the motion. When Mrs. Chamberlain asked her to be president of the Scottish branch it had been hoped that a meeting might have been held in Edinburgh, but owing to the death of her Majesty Queen Victoria it had been postponed by the committee's advice; so that the Scottish branch was at present rather like a soul without a body. She hoped that it might be possible to inaugurate it by a meeting in Edinburgh, which might be addressed by Mr. and Mrs. Chamberlain. All the provosts of counties had been written to by herself and Mr. Gordon, and a good many subscriptions had been received—some from steamship companies, employers of labour, and merchants. Lord Strathcona was a warm supporter—he had given a hundred guineas. She felt the great importance of keeping up the standard of the nursing; that was why so few nurses had as yet been passed on to the committee. It was hoped, however, that a letter in the Scotch papers would bring in more applicants. Scotland had supplied two fully-equipped hospitals for sending to South Africa, so there must be plenty of good material in the country. The idea of the Association was so splendidly simple that it was wonderful how long England had done without it.

Mr. Winston Churchill, M.P., also supported the adoption of the report. He said that he had been specially asked to speak about the question of sick pay for nurses of the Association. There were two objections to affiliating with existing institutions—namely, the nurses themselves objected to paying into a sick fund, and the difficulties of collecting subscriptions from nurses abroad would be enormous. Although it was evident that the Colonies should contribute towards such a fund, he thought that the home organisation should help also.

Sir Hubert Jermingham proposed the re-election of the officers, and the Earl of Westmeath moved a vote of thanks to the chair. A vote of thanks to Lord and Lady Londonderry for kindly allowing the meeting to be held at Londonderry House ended the proceedings.

### The University College Hospital Ladies' Association.

THE first annual meeting of the University College Hospital Ladies' Association was held in the board-room of the hospital on Friday afternoon, when a large number of ladies were present. The chair was taken by the Duchess of BEDFORD, and among those present were the Duchess of Devonshire, Lady Belper, Lady Maple, and Mrs. Asquith. The substance of the report, read by the hon. secretary (Mrs. Bradford), was as follows :—

The University College Hospital Ladies' Association was started in October, 1900, in order to assist the charity by undertaking to provide the special clothing required for the use of patients during their stay in the hospital, and in cases of necessity to supply patients on their discharge with such garments as were more urgently needed.

The Association is very grateful to her Grace the Duchess of Bedford for kindly undertaking the duties of president. There are at present 233 members; of these 21 are vice-presidents, 100 honorary, and 112 working members.

Since its foundation the Association has been principally engaged in equipping the wards and operating theatres with the clothing demanded by modern hospital requirements. This was more especially necessary at the present time owing to the re-building and enlargement of the hospital.

The Association has so far provided 1,094 articles of clothing; 467 of these have been made by a working party organised by Mrs. Frederick Roberts and Mrs. Radcliffe-Crocker, who also undertook the cutting out. The meetings of the working party were held, through the kindness of Mrs. Frederick Roberts, at 102 Harley Street, on alternate Thursdays from November to March. The average attendance of members at its meetings has been 27.

Members of the Association have most generously contributed 381 garments, in addition to many gifts of old clothing (311 articles in all).

The committee wish gratefully to acknowledge donations received in money and materials from ladies and gentlemen who have not become permanent members of the Association.

Provision has been made by the matron for storing and distributing the garments received, and each one is stamped with the die of the Association. The sum of £160 9s. 6d. has been received in subscriptions and donations since the foundation of the Association in October, 1900.

The reliable income of the Association, consisting as it does at present of 100 honorary and 112 working members, will be approximately £66 per annum.

The Committee would be glad if members of the Association would use their best endeavours to obtain gifts of magazines, illustrated papers, and flowers for distribution in the wards; such gifts should be sent to the Ladies' Association, care of The Matron, University College Hospital, Gower Street, W.C., and should have the name and address of the sender firmly fastened inside.

Although the primary object of the Association is to provide clothing, it is hoped that it may ultimately be enabled to promote the welfare of the hospital: (1) By making its wants more widely known ; (2) By the collection of funds to maintain a bed ; and (3) By assisting the charity in other ways; and to effect these objects the Committee trust that during the current year branches of the Association may be established in the provinces, and especially in the counties nearest London, which send large numbers of patients to the hospital.

Lady RUSSELL REYNOLDS moved the adoption of the report; she thought the association might well congratulate itself on the work already done; they owed sincere thanks to the hon. sec., Mrs. Bradford, for the interest she threw into the undertaking. After referring to the new buildings given by Sir J. Blundell Maple, Lady Reynolds said that the association's work appealed to the inner life of the hospital, and by helping the nursing staff they were also helping the medical and surgical staff. In spite of the many and various appeals being made to the generosity of the public, she thought they might look forward with courage and hope to the future.

Speaking in reference to the balance sheet the TREASURER (Mrs. R. J. Godlee) said that £107 was in hand to meet the necessities of the next eight months. The amount of money spent in material, which appeared in the balance sheet, represented only part of the whole, since a good deal had been received in kind. The first year's income was largely composed of donations which they had no right to look forward to again, and it was necessary to make an appeal for funds. It was suggested that all former University men should be written to, and also that branches might be established in the near counties. The kind responses already received encouraged them to go forward. It was true that the whole thing might be done by one person with a mere stroke of the pen, but there was great value in associative work.

Miss EMMA GOLDSMID seconded the adoption of the report, which was carried unanimously.

Lady BELPER moved the re-election of the executive committee—Mrs. Frederick Roberts, Lady Barlow, Mrs. Arthur Barker, Mrs. Carey Foster, Mrs. Henry Lucas, and Mrs. Tuke; this was seconded by Lady Maple, and carried unanimously.

Miss HAMILTON, matron of the hospital, said she was very glad of the opportunity of thanking the members of the Association for their help in providing garments for the patients who were in need of them; shirts, night-gowns, jackets and dressing-gowns were particularly acceptable. When the scheme was started they had nothing in stock for this purpose; the garments were especially valuable to patients going to Convalescent Homes. Men's and boys' clothing was most urgently wanted. The Association would be of very great use in making the wants of the hospital more widely known.

A vote of thanks to the Duchess of Bedford, who thought their thanks were due to the hospital authorities for the use of the board-room, closed the meeting, which adjourned to the nurses' new sitting-room for tea and coffee. This is a large many-windowed room, coloured in a pale quiet green, and here the sisters and nurses dispensed tea to the ladies of the Association. The same quiet green predominates in the bedrooms—the numbers run up to 90—on either side of the corridors, and, as one of the nurses remarked, a very little decoration produces a pretty effect on these bright little rooms. The floors are of blocks, with a rug or a mat over them, and are therefore very easily kept clean by scrubbing. All the nurses are now in the building, and they are certainly to be congratulated on having such dainty little rooms. On Friday they were gay with flowers, and looked most inviting.

### Death in Our Ranks.

WE regret to hear of the death, from rheumatic pericarditis and pleurisy, of Miss Louisa Grace Coupland, a probationer at Ham Green Hospital, Bristol. She was only twenty-two years of age, and had completed eighteen months of her two years' fever training.

### Wants and Workers.

I HAVE promised a set of flannel bed-jackets to a new cottage hospital, and I shall be glad if some nurse would let me know which pattern is the most useful for ordinary cases.—H. M. G.

### South London District Nursing Association.

THE annual meeting was held on Saturday afternoon, at Glenbrook, Nightingale Lane. The large drawing-room was well filled with friends and supporters, and the guests were warmly welcomed by Mr. and Mrs. Brooks. Mr. W. H. DICKINSON, hon. secretary, said that this association was one of the pioneers of district nursing; it began seventeen years ago. It was carried out according to the lines laid down by the Metropolitan and National Nursing Association: only skilled nurses were employed, no relief was given, and it worked in co-operation with existing local organisations. No fees were asked for, but if patients could afford to give they were encouraged to do so. There were now 9 nurses and the lady superintendent at the home in Marmion Road. The number of cases had gone up enormously; from being 377 in the second year, they had risen to 1,475 in 1900. The number of visits paid by the nurses was 27,457. The expenditure was about £800, subscriptions and donations amounted to £561. He thought that drawing-room meetings were the most effective means of raising funds. This was a cheap charity; a little more than 10s. 6d. for each case covered the cost of the nurses' work, and the visits worked out at about 6d. or 7d. each, on the average.

Lord CHELMSFORD dwelt on the usefulness of a society with which he had been for some years connected, which provided dinners for convalescents; it was found of very great use after the early stages of recovery were past; fourteen dinners were given, and they might extend over a month if required.

Lord STAMFORD said he approved of a charity which sought to obtain funds by direct means; he had long set his face against indirect means; he would appeal rather to a sense of duty than to selfish motives. The sense of sympathy and the wish to relieve suffering were adequate in themselves if boldly appealed to. He approved, too, of the co-operation between this association and existing local agencies; he noticed the names of two local Boards of Guardians in the subscription list. He had for many years been in more or less close connection with the Charity Organisation Society in London, and he was, therefore, pleased to see that co-operation existed between the Association and the local branches of this Society. The Nursing Association was an educational agency. He was rejoiced to read, in one of the "specimen cases" in the report, how the nurse had found time to teach a patient's daughter to make various light dishes to tempt her father's appetite. He then alluded to the great importance of the nurses' work in the Board Schools. A great many cases of disease were by this means arrested in the early stages, and the sources of disease were cut off at the fountain head. Most of the minor complaints originated in dirt and disorder, and to arrest them was a very important function. An American who was studying social questions in England had remarked on the almost total absence of agencies "to prevent a man from falling down," though he found plenty to help him when he was down. This was just such a preventive agency.

Dr. HAWARD, consulting surgeon of St. George's Hospital, spoke very emphatically on the same subject. He said that a large proportion of cases of ophthalmia and skin disease would be preventible if treated in the early stages. A skilled nurse recognised the symptoms in children; and if the case was one requiring to be isolated, she was able to see that this was effectively carried out. It was a department of the nurse's work very highly commended by the Government inspector; it was of enormous value to the community at large.

Canon ERSKINE CLARKE, Chairman of the Association, said the work in the schools was a new departure, and, from his own observation, he could say that it was a very useful one.

Miss ALICE HUGHES also spoke of the School Nurses' work, though from a different and original point of view. She was glad to have the opportunity of saying something in support of an institution which thoroughly deserved all the interest and support it could get. Until within the last few years she had herself been a district nurse, and she had also had the responsible work of training others. She was quite delighted to hear Mr. Dickinson's mention of ancient history; an old institution should not be forgotten in the hurry and rush of modern life. It was because of the high principles laid down at the beginning that so much success had been attained. Those present knew the value of a good nurse; how much she did by knowledge, by loyalty, and by obedience. Not only did she help to cut short a great deal of illness, and keep many homes from being broken up, but one of the great factors in district nursing was its educational value. At the very beginning Miss Nightingale spoke of nurses as "health missionaries." Some people objected to the School Nurse's work because it did not take up the whole of her time, and she was able to visit patients perhaps once a week as well. But the results of these isolated visits spread even further than if they were daily ones; they reached that part of the community who were a little apt to be overlooked—the chronic and the old. Young people often thought that the workhouse was good enough for their grandparents, when by a little knowledge—how to prevent bedsores, &c.—the old people could be cared for at home. She thought that it would do much to prevent the tendency which existed to neglect the aged.

Votes of thanks to the speakers, and to Mr. and Mrs. Brooks, brought the meeting to a close, and the guests adjourned to tea and to enjoy the beauties of the garden.

### Appointments.

BURTON-ON-TRENT GENERAL INFIRMARY.—Miss Bessie Butler has been appointed matron. She was trained at Bristol General Hospital, where she was afterwards sister for five years. She has also been night superintendent at Swansea General Hospital, and assistant matron at Poplar and Stepney Sick Asylum.

CHELSEA INFIRMARY.—Miss Minnie Snell has been appointed night superintendent. She was trained at the Grosvenor Hospital for Women and Children, where she was afterwards made charge nurse. Since then she has held the post of charge nurse at Chelsea Infirmary.

JUBILEE HOSPITAL, WOODFORD,—Miss Annie M. Yarrow has been appointed matron. She was trained at the Royal Southern Hospital, Liverpool, and has since been in charge of Jubilee O.P. wing Royal South Hants Hospital, Southampton, sister of women's wards and deputy matron Stanley Hospital, Liverpool, sister of men's medical wards, operating theatre, and casualty department, General Hospital, Swansea, and sister of wards and deputy matron, General Hospital, Weston-super-Mare.

KINGSWOOD SANATORIUM, DELAMERE FOREST. — Miss Isabel D. Wright has been appointed matron. She was trained at the Royal Infirmary, Liverpool. She was subsequently engaged in district work at Kirkdale, and has lately been matron at the Liverpool Cancer and Skin Hospital.

ROTHERHAM HOSPITAL.—Miss Hickman, who was recently appointed sister to the women's and children's wards, has been transferred to the male wards. Miss M. A. Hay has been appointed sister of the women's and children's wards. Miss Hay was trained at Leith General Hospital, and has since been charge nurse at Dumfries and Galloway Hospital, and temporary charge nurse at Chesterfield Hospital.

SALISBURY INFIRMARY.—Miss Louisa O. G. White has been appointed sister. She was trained at Dundee Royal Infirmary, and the "Mary Hewetson" Hospital, Keswick. Subsequently, she has been staff nurse and theatre nurse at Dundee Infirmary.

YORK FEVER HOSPITAL.—Miss Edith Haspell has been appointed matron nurse. She was trained at Kendal Fever Hospital, and has since been nurse at the City Hospital North, Liverpool, charge nurse and assistant matron at Yoker Fever Hospital, near Glasgow, and matron of Kendal Sanatorium.

### The Nursing Question in Irish Poor Law Infirmaries.

REMARKABLE EVIDENCE.

LAST week the Judicial Committee of the Irish Privy Council met in the Council Chamber, Dublin Castle, to consider petitions from several boards of guardians throughout the country, requesting that the Lord Lieutenant in Council should withdraw his approval of the order of the Local Government Board, of February 4th, 1901. As already explained in our columns, the question involved was the right of the Irish Local Government Board to insist upon the appointment of qualified nurses and attendants in Poor Law Infirmaries and Hospitals. In the result the Committee announced that they must advise the Lord Lieutenant to disallow the order as it stands. But they report that evidence has been given establishing to their satisfaction the necessity for some rule or order which will make better provision for efficient nursing. We append some of the more important evidence:—

Dr. Edward C. Bigger, Local Government Board Inspector, said he had been for a number of years visiting medical officer to the Belfast Workhouse. In the Armagh Workhouse at present there were one day nurse, one night nurse, and one temporary nurse. The nursing staff at present, in his opinion, was not sufficient. He had been engaged in inspecting the workhouse infirmaries throughout Ireland. He regretted to say that the nursing staffs were manifestly insufficient and inefficient. In some of the hospitals he had visited there was no trained nurse whatever. There had been very great difficulty in getting the guardians to appoint efficient nurses. There were twenty-eight workhouse Infirmaries in which there was no trained nurse whatever. There were forty or forty-five in which the staff was manifestly insufficient. Among the workhouse infirmaries in which there was no trained nurse were Ballymoney, Bailieborough, Cootehill, Dunfanaghy, and Milford. In Downpatrick there was no night nurse for 112 patients; in Enniskillen there were 58 patients and no trained nurse; in Castlederg there was no trained nurse; in Omagh there were 101 patients and no trained nurse. Among the infirmaries with insufficient nursing staffs were Antrim, with 77 patients and 3 nurses, there being no night nurse; Larne, with 71 patients and only 1 fully-trained nurse; Lurgan, with 182 patients and 2 trained nurses. The proper proportion was one nurse to eleven or twelve patients. For acute cases in general hospitals the proportion was higher. In Belfast Workhouse at present the proportion was about one nurse to fourteen patients.

In cross-examination, Dr. Bigger said that if he were the medical officer at Armagh he would insist upon one or perhaps two additional nurses. He was not aware that the guardians were willing to appoint one additional nurse. In the Belfast Workhouse they were now acting upon the proper principle; he knew no other Boards of Guardians that were. For the last couple of years Belfast was recognised as a training school and their nurses were employed all over Ireland as trained nurses. They were making their nursing staff up to the standard that would be recognised in any well ordered hospital.

ANOTHER INSPECTOR'S EVIDENCE.

Mr. Agnew, Local Government Board Inspector, said that in 1893 he called attention to the fact that there was no night nurse in the Armagh Workhouse. In January, 1894, an inquest was held on the body of an inmate. In that year witness reported that there was only one paid nurse for 60 patients and 23 female lunatics. He discovered that the meat supplies for the week were supplied at the same time and put in pickle. After that an arrangement was made by which the meat should be delivered twice a week and supplied fresh to the patients. In 1895 the Local Government Board sent a recommendation that a night nurse should be appointed, and that night nurse was not appointed until 1898. On February 21st, 1895, he reported of the Armagh Workhouse Infirmary that there was only one nurse for 64 patients and 19 female lunatics. There was no night nurse, and in cases of grave illness or cases in which death was imminent some inmate was told off to attend on the sufferer.

Extracts from reports made by Mr. Agnew in 1896 and 1898 were then read. In September, 1898, he reported that there were in the infirmary 94 patients, of whom 44 were lunatics under the charge of a head nurse and probationer, two paid assistants, and five healthy female paupers. The patients objected to the services of those women, on the ground of their character. On the day of his visit there were four typhoid cases, under the charge of one trained nurse assisted by a pauper attendant.

Mr. Agnew, in reply to questions by counsel, said that, in his opinion, the nursing staff of the infirmary was not sufficient; that the Local Government Board made continuous and repeated endeavours to have it increased, but without avail, and that, speaking generally, the same state of matters existed in almost all the unions in the North of Ireland. He gave several instances, such as the case of Larne Workhouse, where the regular nurse got ill, "and women of doubtful character were placed in charge of the sick wards," and that of Banbridge where "it took three years to get the guardians to appoint a night nurse. Mr. Agnew declared, in conclusion, that it is absolutely necessary for the Local Government Board to possess the power of directing pauper attendants to be appointed, and that it had been a constant uphill struggle to get the guardians to carry out the suggestions of the inspectors.

FORMER INSPECTORS.

Mr. W. L. Micks, a member of the Local Government Board, and a former inspector, said that he used to urge the guardians to appoint a trained nurse, but they thought that idea a "fad," and appointed a farmer's daughter, who had never seen the inside of a workhouse. The Board refused to sanction the appointment, but the guardians insisted on maintaining it. The farmer's daughter had never seen the inside of a hospital; she had no training outside, and had been doing household work all her life. This was not an unusual case. He had charge of twenty or thirty unions, and there was not a single trained nurse in one of them. There was hardly any union except that of Belfast in which the number of nurses approximated to what the Local Government Board deemed necessary. The Board laid a case before counsel, who advised them to issue the Order of February 4th, 1901. The question of nursing was a growing one, and the attitude of the guardians with respect to it was entirely due to want of perception of the necessities of the case, and to a desire to save the rates. On one occasion in a workhouse hospital where there was no night nurse, and where most of the persons in the ward were bed-ridden, a man rolled out of his bed and was found dead in the morning.

Sir George O'Farrell stated that he had been a Poor Law inspector, and was now head of the Lunatic Poor Law Department. He retired from workhouse duties in 1885, but in his present capacity he visited every union in Ireland. His experience was that there was a very great want of trained and skilled attendants on the sick and infirm poor in Irish workhouses. It was quite necessary that a power of directing the appointment of attendants should be vested in some authority.

MEDICAL WITNESSES.

Dr. Smith, medical officer of Naas Union, said he had studied the subject of nursing, and had visited the unions of Limerick, North Dublin, Ennis, Rathkeale, Tipperary, Carlow, Ennistymon, and Corrofin, and he considered that the provision for nursing was deficient in both quantity and quality. Nursing in the modern sense was not appreciated by local boards. But skilled nursing was an absolute necessity for the treatment of poor workhouse patients, and was even more important than medical treatment in a large number of instances. He found it very difficult to get guardians to appoint additional nurses, and the pressure of medical officers led to unpleasantness. There was always a minority of reasonable men, but they were overborne by a majority in a full board called in when such appointments had to be discussed; and the matter was then dealt with by men who knew absolutely nothing of the matter and who came mainly for the purpose of keeping down the expenses. Pauper nurses took their meals and slept in the wards, which was most objectionable.

Sir William Thomson, ex-president of the Royal College of Surgeons, Ireland, said that there should be six attendants for every sixty persons in an ordinary Irish workhouse hospital, and that half of them ought to be fully-trained nurses. Any system that did not provide for the continuous attendance of a trained nurse night and day must be defective.

Sir Norries Cruise, consulting physician, Mater Misericordæ Hospital, Dublin, concurred in the evidence of Sir William Thomson, and added that while ordinarily speaking in a workhouse infirmary with an average of thirty patients in a ward, one trained nurse and three intelligent probationers undergoing training might be sufficient, in the case of an operation, or an epidemic, there would be necessity for further aid.

The evidence for the petitioning unions consisted exclusively of the testimony of members of Boards of Guardians, one of whom complained that his board "was obliged to dismiss a probationer and appoint a trained nurse in her place." Another declared that the guardians of a union were better qualified than the Local Government Board to judge of the requirements of the patients. No attempt was made to dispute the evidence as to the needs of better provision for efficient nursing.

### Everybody's Opinion.

[Correspondence on all subjects is invited, but we cannot in any way be responsible for the opinions expressed by our correspondents. No communication can be entertained if the name and address of the correspondent is not given, as a guarantee of good faith but not necessarily for publication, or unless one side of the paper only is written on.]

### THE NURSES' CO-OPERATION.

"L. M. G." writes: I do not know if any of the other nurses belonging to the Co-operation have written to thank you for your kindness to us. But I and several of my friends thank you for your articles, and for pointing out misstatements in different letters and paragraphs.

"C. W." writes: May I be allowed to make this suggestion—that all new members who wish to join the Nurses' Co-operation should pay an entrance fee, say £2 2s. This would enable the Co-operation to cope with their present difficulties, and place the institution on a sounder basis. I myself would not mind paying a premium.

### "THE GUILTY FEW."

"A Matron" writes: It is quite possible that "A Somersetshire Nurse" has sent stamps in answering advertisements, and has not had replies; but in asserting that the cost of some advertisements are probably more than covered by the stamps sent by nurses she is guilty of an exaggeration; for were it so, it would be necessary for there to be an applicant for every word, and not less than 36 applicants, each sending a stamp, and in very long advertisements, 100 to 200, and even 300, would be required. It is surprising how few nurses send stamps even where a photo requiring a large envelope is expected to be returned to the nurse.

### A WARNING TO MATRONS.

"A. B." writes: I should like to warn matrons of nursing homes against a very successful impostor, calling herself a nurse of the Army Nursing Reserve, and claiming relationship to Miss Gordon, St. Thomas's Hospital. She has succeeded in borrowing money in different ways, by clever strategy, without actually asking for it, from several nursing homes on the South Coast during the last week. She professes to be desirous of getting a relation or friend into a home, and enters into minute details with the perfect experience of a nurse and most carefully discusses all arrangements for the patient to come in the following day. During the interview she remarks that she has lost her ticket and has not sufficient to get back to town, and this is so well done and she appears to be so thoroughly sincere that she has had money offered her on several occasions and by people who are not easily taken in. She is tall, slight, very dark and without teeth—these are probably movable. Age about 35. I trust that this warning may be of timely service to anyone upon whom she may again try her importunities.

### THE BALLOT LIST OF THE GENERAL COUNCIL OF THE ROYAL BRITISH NURSES' ASSOCIATION.

"An Interested Midwife" writes: I enclose voting paper of the R.B.N.A. and wonder how it is that in view of the wishes of so many public-spirited women interested in the Midwives' Bill, there is no matron, so far as I can see, or sister representing midwives on the past or present Council. I also note that Mrs. Strong, of the Glasgow Royal Infirmary, has withdrawn her name. But why? With eleven hospitals, institutions, cottage hospitals, &c., each with a representative of its special hospital, it seems strange to see no maternity hospital mentioned. Surely there must be one R.B.N.A. in such a post in London?

[The full representation of all interests and all opinions on any council is a difficulty, but as a general rule it may be stated that where any opinion is well voiced in the general meetings of a society it will before long obtain representation on the council.—Ed. Hospital.]

### HOMES FOR INEBRIATES.

"Sister in Charge" writes: The description of life in a home for inebriates, published in your issue of April 27th, labours under distinct disadvantages. (1) The writer could have no real experience of the work she attempted to describe—temporary charge cannot make an opinion worth the giving of it; had her charge been one of years she would have apprehended that it is result which gives real interest to the treatment; of this nothing is said. (2) The writer ignores the fact that the whole being is deteriorated and disorganised by inebriety—body, soul, and spirit. It is quite true that the higher nature is in almost all cases reached through the lower; that is to say, all due physical and moral remedies and helps should be used for the renovation of body and soul; but cycling, cards, tennis, needlework, books—not even music—are due remedies for the sin-sick spirit. Here, in this house, where the outward life is in nearly every respect very much the same as that described by your correspondent, we do our utmost to bring home to our charges the great fact that God made us for Himself, and that we can only find our real strength and rest in Him. The result is most happy. In talking on the subject of the treatment of inebriates a short time ago with Sir Dyce Duckworth he begged me to use his name as that of a physician who recognises emphatically that the only treatment likely to lead to a hopeful and permanent good result is that which takes into account the threefold nature of our being. My experience of this difficult, arduous, but if duly done, hopeful work has extended over several years.

### POST GRADUATE COURSES FOR NURSES.

"The Author of the article on 'Nursing in the Erie County Hospital, Buffalo,'" writes: I beg to thank you for publishing my first attempt at writing. So many doctors and nurses are constantly inquiring about the American hospitals that I thought it worth while to send you the article, not daring to hope that it would appear in print. Being on night duty, with a sick patient, I was unable to write sooner, but would very much like to have the subject of Post Graduate Courses for Nurses introduced through your columns. According to my idea a nurse should keep up to date with her work, and a fully-trained nurse does not care to enter as probationer. Perhaps it might be possible for fully-trained nurses to take a third-year course; that is, to undertake the duties similar to those of the third probationary year, or to occupy the position that staff nurses now hold with a smaller salary. The Post Graduate and Polyclinic hospitals in New York take fully-trained nurses for an additional six, nine, or twelve months, which gives nurses who were trained five or ten years previously an opportunity to refresh their memories and get a fair knowledge of new methods and ideas which were not in vogue when they were trained. I notice many advertisements requiring nurses fresh from hospital; should that state of affairs continue or grow, there will be small chance for nurses of long standing to get positions either in hospitals or homes unless the Post Graduate course can be introduced. My American diploma dates 1898, though previous to going abroad I had mental training for two years in Rainhill Asylum, 1891 to 1893, and in the S.M.D. Schools, Herne Bay, 1889 to 1890 (one year), with six months in the American Army. You will consider my experience varied; still, I should like to take a Post Graduate course, at least every five years, if such a thing were possible; and so far as I can learn, there are at present no hospitals in this country where such an advantage is open to nurses.

### AN OPEN-AIR BABY.

"AN ENGLISH NURSE in Florence," enclosing the photo of a very healthy fat baby who, devoid of covering, is taking an air-bath in the corner of an armchair in the open beneath the leafless trees, writes: This child has been brought up in a somewhat unusual manner. Although the climate is very similar to that of England, a few days after its birth it was put out in its cradle in the open air, remaining there the whole day. During the first two weeks it was fed every two hours, but was accustomed from the first day to sleep six or seven hours through the night without being fed. This is an important point for every child, as the digestive organs thus have time to rest. From the second till the tenth week the child was fed every three hours, after which every four, but never during the night, and soon it began to sleep ten to eleven hours straight away. It has never been in a room with closed windows, not even in the night, and during the day is always out of doors. If the weather is warm no harm can come to the child, but if it is cold and during the winter, sometimes below freezing point, the child is watched carefully and so well covered that its body is always perfectly warm. To ensure this it is put into a flannel sack, leaving only the head exposed. The sack is long and large, so as to give perfect freedom to the little arms and legs to kick and move. In the cradle a hot-water bottle is placed near the child's feet, and if necessary one under the mattress. On very cool days a woollen shawl is laid on the pillow, around the child's head. The principal object is to keep its mouth and nose free to breathe in the good air. Every morning before its meal the child gets a cold sponging. In the afternoon, before the warm bath and if the weather is mild, it is often put for about five minutes into the open air or into the sunshine for an air-bath. The child has not been unwell for one minute since its birth, nor has it ever been awake for half an hour during the night. It can remain out of doors safely from before sunrise till after sunset. When six months old it was weaned gradually, being given goat's milk, which is excellent and for many reasons preferable to cow's milk.

### "The Hospital" Convalescent Fund.

THE hon. secretary acknowledges with many thanks the receipt of 5s. from E. A. H., and of 1s. 8d. from Miss S. C. Borgaes.

### The Nurses' Bookshelf.

"BURDETT'S OFFICIAL NURSING DIRECTORY, 1901." A Directory of Nurses, compiled and edited, with the assistance of a small committee of medical men and matrons, by SIR HENRY BURDETT, K.C.B. London: The Scientific Press, Limited, 28 & 29 Southampton Street, Strand. Price 3s.

THIS year's *Burdett's Official Nursing Directory* appears in a new form, and one which we believe will insure for it a great increase of popularity. After much consideration the Editorial Committee came to the conclusion that it would be to the general advantage of those who use the *Directory* to have it in a more compact shape, and at a cheaper price. They accordingly determined to separate from it the detailed account of the principal nurse training schools in the United Kingdom, the Colonies, and the United States of America, which had hitherto formed an introduction to the *Directory*, and to publish the work by itself. It is now, therefore, as the *Nursing Directory*, pure and simple, that it makes its appearance, and in addition to the reduction of two shillings in price, the change to a more convenient size will, doubtless, be widely appreciated. There is no need to dwell on the utility of an official *Nursing Directory*. It has become an absolute necessity, not only to trained nurses, and to members of the medical profession, but also to the community at large. Its value, we are glad to say, increases year by year, and on this occasion a large number of new names have been added. But nurses have still the power to materially augment the usefulness of the book, if only those whose names are not included in it will take the trouble to send the requisite details to the Editor. It is in their interest that we once more suggest this to them. As it is observed in the preface, "During the war in South Africa the particulars of the training and career of those nurses who have gone to the seat of war have been given, whenever available, in the pages of THE HOSPITAL, and the fact that in some cases no particulars were given caused inquiry as to the seeming discrepancy." The explanation is that no information concerning the latter could be obtained, and the same state of things continues to prevail. While, therefore, we are rejoiced to learn that the articles published in our columns last year urging upon nurses the importance of taking steps to have their names entered in the *Nursing Directory* were again not without result, we are hopeful that the day is not far distant when it will be possible for us to ascertain at once, by reference to its pages, the particulars of the career of any nurse who receives an appointment, instead of confining ourselves, in the absence of them, to a bare record of the event. The omission of a nurse's name is, of course, serious to her from other points of view, and as inclusion involves little trouble and no expense, it is difficult to understand why any nurses care to run the risk of the financial loss which exclusion must almost inevitably entail. It may be mentioned that the *Nursing Directory* contains an opening chapter on the principal laws affecting nurses, and a classified list of the various training schools, of which fuller particulars will be found in *The Nursing Profession*.

### TRAVEL NOTES AND QUERIES.

LUCERNE PENSIONS (Lucerne).—On the Gütsch, which is high up at the west end of the town, the Hotel-Pension Wallis will take you from about 7 fcs. per day, and on the Hill of Gibraltar there are two very reasonable Pensions, the Villa Britannia from 7 fcs. and the "Suter" from 6 fcs. How would you like to go to Brunnen, I prefer it to Lucerne, and it is cheaper. It is on the Axenstrasse about half way to Fluelen; Hotel Pension Hirsch will take you for 6 fcs. Gersau is another nice little place on the Lake; Hotel Pension Beau, sejour from 4½ fcs.

A MONTH IN SWITZERLAND (Lemloh).—Second class return to Thun is £5 8s. It is a charming little place, but rather warm for summer. If you stay there go to the Schweizer hof, 5 to 7 fcs. It cannot be called among the mountains. Grindelwald is really on them, but it is very crowded in the late summer and autumn. Go to Hotel-Pension Grindelwald, pension from 6 fcs. Wengen is a delightful spot still higher. Pension Wengen from 6 fcs. Interlaken is hot and crowded. Lucerne, Meyringen Grindelwald or Wengen would make a charming little tour. Brienz and Thun can be seen from Meyringen or Grindelwald £6 5s. would cover the journey. From Lucerne to Meyringen you cross the Brunig Pass.

### Echoes from the Outside World.

### AN OPEN LETTER TO A HOSPITAL NURSE.

THE decision of King Edward that May 24th is still to be observed in memory of Queen Victoria, will cause much satisfaction in the Colonies. In England, although the King has now ordained that the date of the late Queen's birthday shall be observed as a holiday in the Government offices, we have never made it either a national or a Bank holiday, but in many of the Colonies it has always been looked upon as almost the most festal day in the year, and it would have caused much inconvenience, as well as considerable disappointment, to schoolboys and schoolgirls in particular, if a day which ever since they could remember had been a holiday had suddenly ceased to be treated as one. For the future it will probably be called Victoria Day, and the trooping of the colours is likely to be transferred to the anniversary of Coronation day which will, as at present intended, fall in the summer. This is the more desirable because the King's birthday proper falls in November, an inconvenient date for any outdoor functions.

---

ONE of the reasons why our English Royal Family are so popular is because they always take such a kindly interest in all with whom they are brought closely into contact. The goodness and the consideration which Queen Victoria showed to all her dependents is proverbial, and the same virtues are characteristic of the King. He, as well as his wife, has always been most thoughtful for the happiness of those who served him whether in London or at Sandringham, but naturally the deeds which, whilst he was only Prince of Wales, could be hidden under a bushel, are now exposed to the full light of day. The touching little incident which occurred last week reveals him in no new light, but it is pleasant to read how a housemaid, having been taken ill at Marlborough House, and ordered away to a private hospital for an operation, was seen by the King just after she had been placed in the ambulance. He was returning from a drive, and alighted at once from his carriage to come and speak to the girl, telling her to keep up her spirits as she would soon be able to return completely restored. His words of happy omen seemed prophetic, for the operation was successfully performed and the patient is well on the road to recovery. Last Saturday Queen Alexandra quite unexpectedly paid a visit to the West Norfolk and Lynn Hospital. She arrived in a motor car attended by Sir Dighton Probyn and the Hon. Charlotte Knollys. Her Majesty spent about three-quarters of an hour in the institution, most of the time being passed in conversation with the patients. Another kindly deed is recorded of the King's only unmarried daughter. The Alexandra Technical School, at Sandringham, in which the Royal folks take much interest, was in need of funds, and the Princess Victoria has sent to the Home Arts and Industries Association—which has an exhibition at the Albert Hall—a beautiful screen. The two decorative panels have been worked by herself, and the money for which it is sold will be given to the school. The design is of large loosely-petalled mauve irises with foliage, and the work has been carried out in fine darning and chain stitch, the Royal autograph appearing in marking stitch. It is mounted in dark rosewood, having at the top stained glass, in which more flowers appear. The whole is a very artistic production, and had it been done by an unknown worker would probably have provoked much favourable comment.

---

THOSE nurses who are of Scotch descent will be especially interested in the proposal of Mr. Andrew Carnegie, the American millionaire, to present the four universities of his native land with £2,000,000 sterling. He is desirous of providing a fund available for all Scottish students, male and female, which will defray all their college fees and thus provide them with a free education. This applies, of course, more particularly to those whose means are limited. Colonial and foreign students will still pay the same as before. Everyone cannot but admire the munificence of the patriotic Scotchman, and his desire that the high reputation which his countrymen and country women have already earned in the literary and educational world should be sustained; but the effect of his scheme can be better appraised when the full details are known. The actual university fees only amount, it is said, to about £9 a head. It is the expenses of living which make it impossible for the student in Scotland whose parents are too poor to send him help from home to embark upon a university career. This difficulty Mr. Carnegie's gift would, apparently, do nothing to overcome, and also it appears that if elementary and university teaching are both to be free, it will be found impracticable to charge for secondary education. However, it need hardly be said that there is no disposition on the other side of the Border to "look the gift horse in the mouth."

---

THE third series of pictures representative of "Picturesque Holland" by Nico W. Yungmann is being exhibited at the Dowdeswell Galleries, 160 New Bond Street. The pictures, which are all frescoes or water-colours—of which the former predominate—are well worth a visit. The colours are wonderful—a wealth of brightness and warmth, and yet so exquisitely toned and worked in that there is not the slightest suggestion of gaudiness. One, "Watching for Dan" (2), is a specially good example of the artist's skill. It is a Dutchwoman, ruddy of hue and tanned by exposure to wind and weather, with a good, earnest face, looking out from a stone pier over the dancing waves. The hair is partly concealed by a close-fitting Dutch cap of muslin and beautiful lace her eyes are shaded by her hand, and there is very much in the picture to attract. "Ave Maria" is a manly boy, about twelve years of age, clad in a red jacket and a brown sort of shirt, kneeling on the floor before an image of the Virgin Mary placed close to the edge of an ordinary kitchen table. The rapt, devoted expression of the lad is cleverly caught. Each picture is to be sold in its frame; and the frames are so unique that they deserve a word of mention. They are of natural, unpolished wood, carved in what I believe is called "chip carving," the design picked out in colours—dull red and dull green, dull blue and metallic tints. The result is particularly beautiful, and the frames enhance the merit of the pictures in a way impossible to gilded surroundings.

---

"SWEET NELL OF OLD DRURY" has attained the 250th representation—a sure mark of the popular favour; and as I had a friend staying with me I thought I could not do better than take her to see "Sweet Nell" at the Globe Theatre. For "Sweet Nell" indeed she is. Miss Julia Neilson acts in the most natural and in the most delightful manner, and looks more beautiful than I have ever seen her. Ill-natured cynics say that one woman never admires another; but that is pure rubbish, and it is quite impossible for anyone with eyes in their head to do other than worship at the shrine of such beauty as Miss Neilson's. It is easy to understand the fascination such a happy healthy creature had for the storm-tossed, weak-minded, Charles II., and that Nell Gwyn could, when she chose, get any favour she wished. The interest in the play is very well sustained throughout, and when Nell has dressed herself in Judge Jeffreys' wig and clothes and is administering justice in a way quite unknown to the tyrannical unjust judge, I could almost have called out to the servant to hasten as he leisurely lighted the ten candles one after the other, for I felt that every moment increased the risk of the plucky girl being discovered and frustrated in her kindly schemes. During the summer months there are to be no performances on Saturday evenings, but there will be matinees on Wednesdays and Saturdays.

### For Reading to the Sick.

WHITSUNTIDE.

"THE FRUIT OF THE SPIRIT IS—PEACE."

I have but Thee, O Father: let Thy Spirit
Be ever with me, to comfort and uphold;
No gate of pearl, no branch of palm, I merit,
Nor street of shining gold.
Suffice it—my good and ill unreckoned,
And both forgiven through Thy abounding grace—
I find myself by hands familiar beckoned
Unto my fitting place.
There, from the music round about me stealing,
I fain would learn the new and holy song,
And find at last, beneath Thy tree of healing,
The peace for which I long.

*J. G. Whittier.*

Grant us Thy Peace throughout our earthly life,
Our balm in sorrow, and our stay in strife!
Then, when Thy Voice shall bid our conflict cease,
Call us, O Lord, to Thine eternal Peace!

*Ellerton.*

The Holy Spirit, as given to the Church, and to each member of the Church, is not an illumination once for all, or a confirmation once for all, but a germ of light and strength capable of indefinite development. It is potentially a revelation of all truth to the intellect, and a communication of all power to the will. It is a growing and expansive force, not a force which exhausts itself in one impulse.

*E. M. Goulburn.*

Pray for light to see yourself as you are; that the Holy Ghost may convince you of sin; that the work begun in Baptism may go on, and grow in you, till you attain unto the measure of the stature of the fulness of Christ; that you may never, by the neglect of the means of grace, quench the Holy Spirit.—*A. G. Mortimer.*

O God, the supreme and only joy of Thy people, in Thee alone my heart finds rest. Lord, if only I have Thee, I have enough. Keep me in peace, and continue Thou Thy mercy towards me. Strengthen me in every trial by the consolations of Thy Holy Spirit; and when the night of death draws near, take me, I beseech Thee, unto the land of perfect and eternal rest, that I may see Thy Face in righteousness, and be satisfied with the joy of Thy presence for evermore.—*From the German.*

O Spirit of our spirit, life's pure Fount!
True Friend of the Bridegroom whom we wait!
Reveal Him clearer to our souls, that mount
With keen expectance towards their promised state.

That when He comes for whom we hourly pray,
And we are one with Him, in heart and mind,
He will unfold to us the wondrous way
In which Thy love and His for us combined.

Till then, we yield ourselves in deepest trust
Into Thy Hands, their impress to receive;
We would adore Thee, humbled to the dust;
O Holy Ghost, we do in Thee believe.

*C. M. Noel.*

### Notes and Queries.

**The Editor is always willing to answer in this column, without any fee, all reasonable questions, as soon as possible.**
**But the following rules must be carefully observed:—**
**1. Every communication must be accompanied by the name and address of the writer.**
**2. The question must always bear upon nursing, directly or indirectly.**
**If an answer is required by letter a fee of half-a-crown must be enclosed with the note containing the inquiry.**

*Homes of Rest.*

(74) Will you kindly give me the addresses of some Homes of Rest for Nurses where a nurse (who is sadly in need of a change after a long illness, and is unable to pay for very expensive lodgings) might benefit by a stay at some bracing sea-side place? I believe there are such homes for nurses and shall be glad, through your kindly help, to know more about them.—*Economic.*

The names of a number of such Homes will be found in "Homes and Hospitals for the Benefit of Gentlewomen" to be obtained at the Army and Navy Stores, Victoria Street, Westminster. Price 1s.

*A Question of Notice.*

(75) I should be glad if you could help me in the following matter:—I was engaged as charge nurse at a salary of £30 a year, plus board and lodging. In the event of my being asked to resign for the reorganisation of the staff, what notice can I claim? When I was elected for the appointment there was no agreement on either side about leaving.—*L. S. D.*

It depends in the circumstances upon how you are paid. If monthly, you can only claim a month's notice; but if quarterly, you are entitled to a quarter's notice. But it is always better to have an agreement.

*Bed Tickets and Temperature Charts.*

(76) We are just now furnishing a new wing to our hospital, and are anxious to know if you could tell us through the pages of your valuable paper which you consider the best and newest method of hanging bed-tickets and temperature charts over patients' beds, and telling us where such appliances can be obtained?—*Matron.*

We strongly advocate the use of metal as against that of either wood or pasteboard backing. A metal back with a clip which will hold a paper firmly is preferable to arrangements with corners into which the paper has to be tucked. With this proviso, you might obtain what you want from any of the surgical instrument makers.

*Hearing.*

(77) What hospital in S.E. London could I attend for my hearing?—*Ellen M.*

The special hospitals are situated W. or W.C., but you can have first-rate attention at Guy's Hospital, St. Thomas Street, Borough, S.E.

*A Grievance.*

(78) Do you think it right that nurses who have only fever training should take the same fees and percentage as nurses who have fever training in addition to the three-year certificate in general nursing? What use is the three-year certificate?—*M. C. T.*

Fever nurses must be allowed to receive the market value of their work—no one will pay more. The three years' certificate is an invaluable qualification, and it opens a wider field of employment.

*Deformities.*

(79) Can you tell me if there is a hospital in or near Liverpool for the treatment of deformities?—*M. H.*

The nearest special hospital is the Royal Orthopædic and Spinal Hospital, Newhall Street, Birmingham. Write to the Secretary for particulars.

*Diet Sheets.*

(80) Is it possible to obtain a diet sheet suitable for a training home of thirty girls? 2. What ought the expenditure per week to be? 3. Can you tell me of a good diet table for an infant?—*A Subscriber.*

1. How would "Hospital Expenditure: the Commissariat" suit you? 2. It will be regulated by local prices: a shilling per head per diem is a fair average, but it can be managed in rather less under favourable conditions with good management. 3. "The Physiological Feeding of Infants" and "The Physiological Nursery Chart," price of each 1s., are what you want.

*Convalescent Home.*

(81) Is there a convalescent home where a young woman suffering from fits (in consequence of shocks) could be received free of charge?—*M. L. Y.*

She had better apply for admission to the National Hospital for the Paralysed and Epileptic, Queen's Square, Bloomsbury, W.C. Fits render a patient ineligible for most convalescent homes, and the National Hospital has its own at East Finchley.

*Child's Nurse.*

(82) Could you kindly give me the address of the institute in London where women are trained as child's nurses?—*E. B.*

The Norland Institute, 29 Holland Park Avenue, London, W.

**Standard Books of Reference.**

**"The Nursing Profession: How and Where to Train." 2s. net: post free 2s. 4d.**
"Burdett's Official Nursing Directory." 3s. net.; post free, 3s. 4d.
"Burdett's Hospitals and Charities." 5s.
**"The Nurses' Dictionary of Medical Terms." 2s.**
**"Burdett's Series of Nursing Text-Books." 1s. each.**
**"A Handbook for Nurses." (Illustrated.) 5s.**
**"Nursing: Its Theory and Practice." New Edition. 3s. 6d.**
**"Helps in Sickness and to Health." Fifteenth Thousand. 5s.**
**"The Physiological Feeding of Infants." 1s.**
**"The Physiological Nursery Chart." 1s.; post free, 1s. 3d.**
**"Hospital Expenditure: The Commissariat." 2s. 6d.**
**All these are published by THE SCIENTIFIC PRESS, Ltd., and may be obtained through any bookseller or direct from the publishers, 28 and 29 Southampton Street, London, W.C.**

